# The Association Between Joint Laxity and Post-Dural Puncture Headache

**DOI:** 10.7759/cureus.41304

**Published:** 2023-07-03

**Authors:** Nezir Yılmaz, Mustafa Çukurlu

**Affiliations:** 1 Department of Anesthesiology and Reanimation, Adıyaman University Training and Research Hospital, Adıyaman, TUR; 2 Department of Orthopaedics and Traumatology, Adıyaman University Training and Research Hospital, Adıyaman, TUR

**Keywords:** intracranial hypo-tension, beighton score, joint laxity, post-dural puncture headache, spinal anesthesia

## Abstract

Objective

This study aimed to investigate the relationship between joint laxity and post-dural puncture headache (PDPH).

Methods

A total of 123 patients with PDPH - 73 females and 50 males - were included in the study. The patients were examined regarding joint laxity and classified into two groups according to the Beighton score. Those with a Beighton score between 0 and 3 were classified as Group I, and those with a score greater than 4 were classified as Group II. Data related to the demographic characteristics of the patients, time of onset of PDPH, severity, need for medical treatment, need for an epidural blood patch, and length of hospital stay were recorded, and a comparison was made between the two groups.

Results

There was no significant difference between the groups in terms of age, gender distribution, and PDPH onset time (p>0.05). In Group II, which included patients positive for joint laxity, total headache duration, headache severity, need for medical treatment, need for epidural blood patch, and hospital stay were significantly higher than in Group I (p<0.05).

Conclusion

Joint laxity may increase the risk of PDPH after spinal anesthesia and may affect treatment processes. The Beighton score can determine the development and severity of PDPH in patients with joint laxity. Assessing joint laxity and Beighton score can improve clinical decision-making in managing PDPH and positively affect patient outcomes.

## Introduction

One of the well-known potential complications of spinal anesthesia is post-dural puncture headache (PDPH). This complication usually occurs within one to five days after lumbar puncture, leading to a clinical picture localized in bilateral, frontal, retroorbital, or occipital regions. Headache may change in severity with changes in position and may be accompanied by symptoms such as sensitivity to light, nausea, and tinnitus. Risk factors for developing PDPH include female gender, pregnancy, young age, type of spinal needle used, its diameter, and the number of interventions [1,2].

In pathophysiology, it is accepted that the main cause of PDPH is cerebrospinal fluid (CSF) leakage resulting from a dural defect at the lumbar puncture site. Intracranial hypotension occurs when CSF loss from the dural defect exceeds CSF production. This causes traction on structures sensitive to pain in the head and neck regions, which can lead to headache and neck pain [2]. In cases of connective tissue diseases and hypermobile joint diseases, it has been reported that weakness in the dural tissue and disorders in the elasticity of the structures surrounding the region contribute to the development of intracranial hypotension and consequently to the emergence of post-spinal headache [3,4].

The Beighton and Horan Joint Mobility Index, commonly known as the Beighton score, is the most commonly used scoring system for assessing general joint laxity. The popularity and widespread use of this tool are attributed to its reliability and easy applicability [5,6]. In our study, we aimed to examine the relationship between joint laxity and the development of PDPH. Primarily, we hypothesized that the increase in CSF leakage due to dural defect in joint laxity may lead to patients developing PDPH. However, we found no study investigating this issue in the literature. Therefore, our study aimed to examine the relationship between joint laxity as determined by the Beighton score and the development of PDPH.

## Materials and methods

This prospective study was approved by the Adıyaman University Non-Interventional Ethics Committee (approval number: 2021-10/25). Between 01.01.2022 and 01.07.2022, patients belonging to the American Society of Anesthesiology (ASA) classification I-II, who underwent spinal anesthesia with a 27 G pen-tipped spinal needle in a single attempt, were identified. The patients were followed up at the sixth, 12th, 24th, 36th, and 48th hours postoperatively and queried about the development of headaches. Our exclusion criteria were as follows: patients with chronic headaches like migraines, those with neurological deficits, allergies to local anesthetics, coagulopathy, contraindications for spinal anesthesia, patients who were under 18 or over 65 years of age, those who received a different spinal needle than a 27 G pencil-point needle, and those who required multiple attempts during spinal anesthesia.

Information such as age, gender, height, and weight of the patients, demographic data, and ASA risk groups were recorded. Before spinal anesthesia was administered, patients were monitored for parameters such as electrocardiography (ECG) test results, heart rate (HR), respiratory rate (RR), blood pressure (BP), and peripheral oxygen saturation (SpO_2_). A 20 G intravenous catheter was inserted through the antecubital region for intravascular access, and a 10 ml/kg/h crystalloid infusion was started. The patients were sterilized with povidone-iodine by determining the intervention area from the L4-5 level in the sitting position. The intrathecal space was reached using a 27 G pen-tipped spinal needle. After CSF flow was observed, the neuraxial blockade was completed with 0.5% heavy 10 mg bupivacaine.

During postoperative pain follow-up, patients with headache complaints were further analyzed. Patients with bilateral headaches affecting the frontal, occipital, and retroorbital regions and whose headaches varied depending on the position were evaluated as suffering from PDPH. Patients with clinical findings of PDPH were informed about the purpose and method of the study. Informed consent was obtained from the patients before their participation in the study.

Joint laxity examination

**Figure 1:**
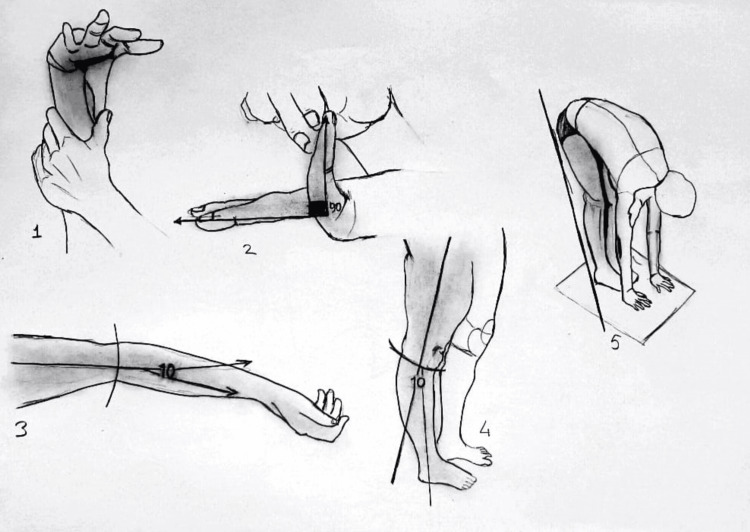
The Beighton and Horan Joint Mobility Index* *[6] 1. Bilateral thumb apposition: thumb contacting the inner surface of the forearm (1 point each for left and right). 2. Bilateral fifth finger extension: at least 90° hyperextension for both sides (1 point each for right and left). 3. Bilateral elbow extension: at least 10° hyperextension for both sides (1 point each for right and left). 4. Bilateral knee extension: at least 10° hyperextension for both sides (1 point each for right and left). 5. Trunk and hip flexion: maintaining knee extension with palms touching the ground (1 point) Permission to use the image has been obtained from the original publishers

Patients participating in the study underwent a joint laxity examination and the Beighton score was used to assess joint laxity. The Beighton joint laxity examination is based on examining five different joint movements (Figure 1). The patients were evaluated on each maneuver they could do, and 1 point was assigned for each maneuver. Scores were evaluated out of a total of 9, and those with a score of 4 or higher were considered positive in terms of joint laxity, and those with a score between 0 and 3 were considered negative.

Management of PDPH

Before starting treatment, headache severity was evaluated in patients with PDPH. The Lybecker classification system was used to determine the severity (Table 1) [7].

**Table 1:** Lybecker's classification of PDPH* *[7] PDPH: post-dural puncture headache; NSAIDs: non-steroidal anti-inflammatory drugs

Mild	Moderate	Severe
Postural headache with slight restriction of daily activity	Postural headache with significant restriction of daily activity	Postural headache with complete restriction of daily activities
Not bedridden	Bedridden during part of the day	Bedridden all day
No associated symptoms	With or without associated symptoms	Associated symptoms present (photophobia, tinnitus, vomiting)
Responds well to non-opioid analgesics (NSAIDs, paracetamol, caffeine)	Requires addition of opiate derivatives	Not responsive to conservative management

In our hospital, a step therapy protocol is used to treat PDPH. Bedrest, oral hydration, and oral analgesics containing paracetamol + caffeine are given to the patients initially (first-stage treatment). Depending on the severity of the headache, non-steroidal anti-inflammatory drugs (NSAIDs) and narcotic analgesics are added to their medication course. If the headache persists after 48 hours, patients switch to the second-stage treatment. At this stage, intravenous hydration, caffeine, and abdominal corset treatment are employed. In addition to using paracetamol + caffeine for analgesia, NSAIDs and narcotic analgesics are added when needed. If the headache persists for seven days, an epidural blood patch is applied. Headache severity, duration of headache, need for medical treatment, and epidural blood patch application status of the patients are documented.

Epidural blood patch

After standard monitoring, intravascular access was provided for emergency drug administration. The intervention area was determined and sterilized with povidone-iodine with the patient in the sitting position. Then, the epidural space was reached by the loss of resistance method by using a Tuohy needle with a thickness of 18 G. Approximately 20 ml of autologous blood was collected from the patient and administered to the epidural area in divided doses of 5 ml.

Statistical analysis

Descriptive statistics of the data [mean, standard deviation (SD), median, ranges, frequency, and ratios] were used. The distribution of variables was measured with the Kolmogorov-Smirnov test. The Mann-Whitney U test was used for the analysis of quantitative independent variables. The Chi-square test was used to analyze qualitative independent variables, and Fischer's exact test was used when the Chi-square test conditions were not met. All analyses were performed using the IBM SPSS Statistics program version 28.0 (IBM Corp., Armonk, NY).

## Results

**Figure 2:**
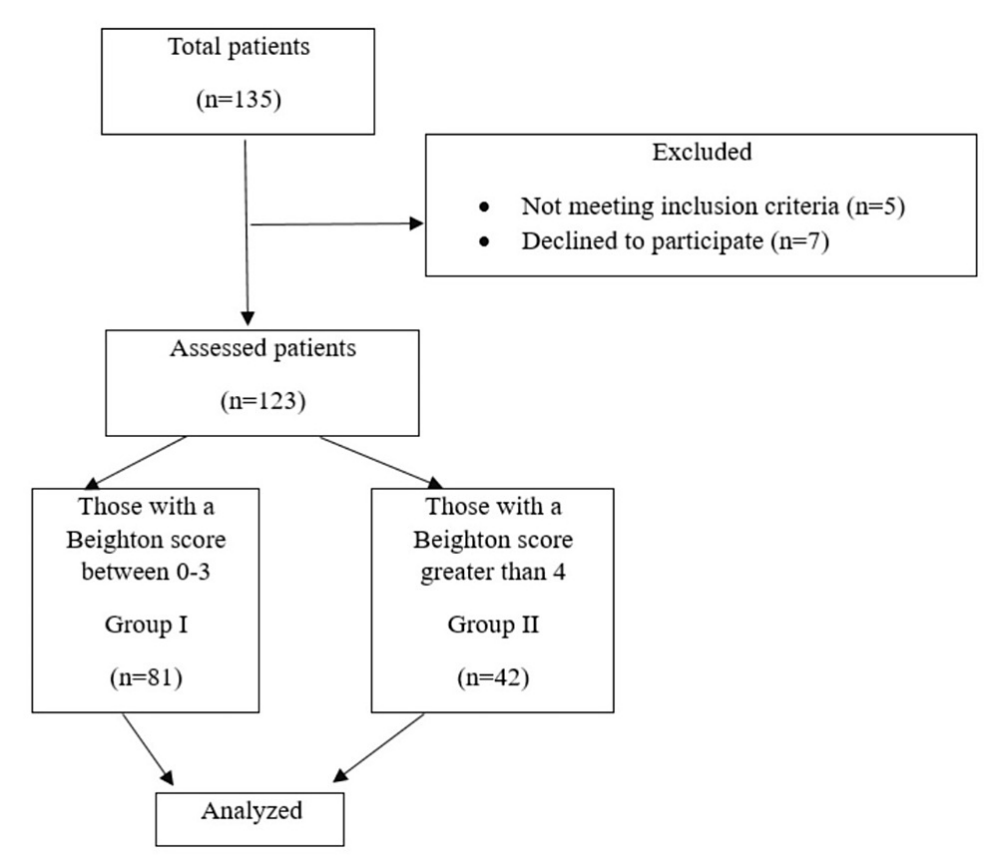
Flow chart depicting the study design

A total of 135 patients who developed PDPH were identified based on the study protocol. Of them, five patients were excluded because they were incompatible with the study protocol in terms of the ASA score and the number of interventions. In addition, seven patients refused to participate in the study (Figure 2). Ultimately, 73 (59.3%) of the 123 patients included in the study were female, and 50 (40.7%) were male. In addition, 42 patients (34.1%) were found to be positive for joint laxity. There were 18 patients (14.6%) who needed an epidural blood patch (Table 2).

**Table 2:** Patient characteristics SD: standard deviation

Parameters		Range/median	Mean ± SD or n/%
Age, years		18.0 – 46.0/27.0	28.2 ± 7.0
Gender	Female	-	73/59.3%
	Male	-	50/40.7%
Beighton score		0.0 – 8.0/2.0	2.7 ± 2.1
Beighton score	≤3	-	81/65.9%
	≥4	-	42/34.1%
Duration of headache, days		1.0 – 9.0/3.0	3.4 ± 1.9
The onset of headache, postoperative	I day	-	68/55.3%
	II days	-	49/39.8%
	III days	-	6/4.9%
Severity of headache	Mild	-	72/58.5%
	Moderate	-	41/33.3%
	Severe	-	10/8.1%
First-step medical treatment		-	123/100.0%
Second-step medical treatment	(-)	-	81/65.9%
	(+)	-	42/34.1%
Epidural blood/serum patch	(-)	-	105/85.4%
	(+)	-	18/14.6%
Duration of hospitalization, days		2.0 – 10.0/3.0	4.2 ± 2.2

There was no significant difference between the groups in terms of age and gender distribution of the patients (p>0.05). Total headache duration in Group II (4.9 ± 2.3 days) was found to be higher than in Group I (2.6 ± 1.1 days) (p<0.05). There was no significant difference between the groups regarding the onset of headache after surgery (p>0.05) (Table 3).

**Table 3:** Comparison of patient characteristics and headache-related data between groups ^X²^Chi-square test. ^m^Mann-Whitney U SD: standard deviation

Parameters		Group I	Group II	P-value
		Mean ± SD/(median) or n/%	Mean ± SD/(median) or n/%	
Age, years		28.4 ± 7.1/(27.0)	27.7 ± 7.0/(27.5)	0.621^m^
Gender	Female	46/56.8%	27/64.3%	0.422^X²^
	Male	35/43.2%	15/35.7%	
Beighton score		1.5 ± 1.1/(2.0)	5.2 ± 1.2/(5.0)	-
The total duration of the headache		2.6 ± 1.1/(2.0)	4.9 ± 2.3/(5.0)	0.000^m^
The onset of headache, postoperative	I day	47/58.0%	21/50.0%	0.673^X²^
	II days	30/37.0%	19/45.2%	
	III days	4/4.9%	2/4.8%	
Severity of headache	Mild	64/79.0%	8/19.0%	0.000^X²^
	Moderate	17/21.0%	24/57.1%	
	Severe	0/0.0%	10/23.8%	

Headache severity was significantly higher in Group II (p<0.05). Also, the need for second-stage medical treatment was found to be significantly higher in Group II (66.7%) than in Group I (17.2%) (p<0.05). The epidural blood patch rate was significantly higher in Group II than in Group I (p<0.05). The length of hospital stay was found to be significantly higher in Group II (5.8 ± 2.5 days) than in Group I (3.3 ± 1.4 days) (p<0.05) (Table 4).

**Table 4:** Comparison of treatment data between groups ^X²^Chi-square test SD: standard deviation

Parameters		Group I	Group II	P-value
		Mean ± SD/(median) or n/%	Mean ± SD/(median) or n/%	
First-step medical treatment		81/100.0%	42/100.0%	1.000^X²^
Second-step medical treatment	(-)	67/82.7%	14/33.3%	0.000^X²^
	(+)	14/17.2%	27/66.7%	
Epidural blood/serum patch	(-)	81/100.0%	24/57.1%	0.000^X²^
	(+)	0/0.0%	18/42.9%	
Duration of hospitalization, days		3.3 ± 1.4/(3.0)	5.8 ± 2.5/(6.0)	0.000^X²^

## Discussion

Meninges are membranous connective tissues that cover the central nervous system and consist of fibers containing collagen and elastin [8]. The dura mater is a fibrous connective tissue with the thickest as well as a hard and dense structure among these meninges. The dura mater offers flexibility that adapts to stretching movements thanks to its elastic fibers, while the taut structure formed by collagen fibers protects the spinal cord. The dura mater is approximately 400 μm thick and has a rich vascular network and extensive neural innervation [9,10].

Procedures such as epidural injections, lumbar punctures, spinal surgeries, and neuraxial blocks can cause iatrogenic (treatment-induced) meningeal injury. Due to the damage to the dura mater in neuraxial blocks, CSF leakage may occur and it depends on the elastic properties of the arachnoid membrane to prevent leakage [11]. Repairing a defect in the dural tissue is a slow and gradual process that can take up to six weeks in some cases. Connective tissues play an important role in this healing process, but connective tissue diseases can negatively affect this process [12].

Joint laxity or hypermobility is an important symptom in soft tissue pathologies [13]. The main cause of joint laxity is genetic factors. The flexibility of ligaments, which surround the joint structure and play an important role in joint movements, is controlled by genes such as collagen, fibrillin, and elastin [14]. However, excessive use of joint movements can also lead to joint laxity, especially in activities such as ballet, gymnastics, and acrobatics. Joint laxity is most common in infancy and childhood and decreases rapidly with age. It is more common in women than men in all age groups [15]. It has been reported that 20% of women and 10% of men have a more flexible joint structure than the average population [16]. In addition, the increase in some hormone levels during pregnancy may affect ligament laxity [17,18].

The Beighton score is a method generally used to measure joint laxity and has also been associated with spinal mobility. In a study by Kim et al., intervertebral mobility evaluated with functional radiographs was significantly higher in patients with a Beighton score ≥4 [19]. Studies conducted in pediatric age groups reported that 35% of the participants had a Beighton score ≥5 [20,21]. In adult patients with shoulder joint laxity and femoroacetabular impingement, it was reported that 34% and 32.7% of the participants had a Beighton score ≥4 [22,23]. Our study found that 34.1% of the participants had joint laxity based on the Beighton score. These values could be attributed to the young age of the patient population, the high number of female patients, and the high number of pregnant patients.

Lybecker, who devised the PDPH classification, reported that in a group of patients who underwent elective surgery, 11% had mild, 19% had moderate, and 30% had severe headaches; 40% were treated with conservative methods [7]. Another study reported that 37.1% mild, 34.3% moderate, and 28.6% severe incidences of PDPH were detected [24]. In our study, 58.5% of the patients who developed PDPH had a mild headache, 33.3% had moderate and 8.1% had a severe headache. The reason for the low rate of severe PDPH and high rate of mild PDPH in our study when compared to the literature is related to using only 27 G spinal needles in patients and including only those patients who had spinal anesthesia in a single attempt.

As reported in the literature, connective tissue diseases, especially weakness in dural tissue, meningeal diverticulum, or dural ectasia may cause spontaneous intracranial hypotension [25,26]. This could lead to the development of symptoms such as PDPH. Significantly, PDPH may occur in individuals with connective tissue disease despite the absence of neuraxial interventions [27]. This suggests that the weakness of the dural tissue due to the underlying connective tissue disease may play a triggering role in the development of PDPH.

Youngblood et al. have reported that the risk of headache development and the need for an epidural blood patch increase after neuraxial interventions in individuals with connective tissue disease. Studies conducted on this topic have shown that the risk of developing headaches after neuraxial intervention increases fourfold, and the need for epidural blood patch increases threefold in individuals with connective tissue disease [28]. Similarly, our study found that the development of moderate and severe PDPH, the need for medical treatment and epidural blood patch, and the length of hospital stay were higher in the group with joint laxity. These results show that connective tissue diseases may increase the risk of complications after neuraxial interventions and affect the treatment process.

This study has a few limitations, primarily its small sample size. Moreover, the grouping of the patients was performed after the diagnosis of PDPH. Also, the majority of the patient population consisted of young pregnant women whose joint laxity had been caused by hormonal changes. Randomized controlled studies involving a more comprehensive joint laxity examination and where grouping is performed in the preoperative period may provide more precise information.

## Conclusions

Joint laxity may increase the risk of PDPH after spinal anesthesia and may affect treatment processes. In patients with joint laxity, there is a greater need for an epidural blood patch, and hospital stays are prolonged. The Beighton score can determine the development and severity of PDPH in patients with joint laxity. Hence, analyzing joint laxity and Beighton score can improve clinical decision-making in managing PDPH and positively affect patient outcomes.
